# The relationship between chronotype and suicidal attempt in patients with schizophrenia

**DOI:** 10.1192/j.eurpsy.2024.1573

**Published:** 2024-08-27

**Authors:** N. Göktürk, P. G. Özdemir

**Affiliations:** ^1^ PSYHİATRİC AND MENTAL HEALTH NURSİNG, VAN YÜZÜNCÜ YIL UNIVERSITY; ^2^PSYHİATRİC AND MENTAL HEALTH, VAN YÜZÜNCÜ YIL UNIVERSITY DURSUN ODABAŞ MEDICAL CENTER, VAN, Türkiye

## Abstract

**Introduction:**

Individuals with schizophrenia are known to be at an increased risk of suicidal behavior (Sher & Kahn, 2019). However, the relationship between chronotype, which refers to an individual’s preference for sleep-wake patterns, and suicidal attempts in schizophrenia patients remains an area of interest and investigation.The relationship between chronotype and suicidal attempts in schizophrenia patients has not been extensively studied. However, research in other populations has shown that individuals with evening chronotypes, also known as “night owls,” may be at a higher risk of mental health issues, including depression and suicidal ideation (Verma et al., 2016). It is plausible to hypothesize that individuals with schizophrenia who have evening chronotypes may also be at an increased risk of suicidal attempts. Further research is needed to explore this relationship and its potential implications for clinical practice. In conclusion, the relationship between chronotype and suicidal attempts in schizophrenia patients is an area that requires further investigation.Early identification and intervention are crucial in preventing further suicidal attempts in this vulnerable population. Future research should focus on exploring the relationship between chronotype and suicidal attempts in schizophrenia patients to provide a comprehensive understanding of the factors contributing to suicide risk in this population.

**Objectives:**

This study investigates the relationship between chronotype and suicidal attempts in patients with schizophrenia.

**Methods:**

The study was conducted cross-sectionally using quantitative research methods and using purposive sampling. The personal information form and scales used for data collection in this study, which was planned with patients hospitalized in the psychiatric ward and patients applying to the outpatient clinic, are based on self-report. The personal information form developed by the researcher by reviewing the literature, the Morningist-Evening Scale (SAM), the Suicide Probability Scale, the Suicidal Behavior Scale, the Positive Symptoms Rating Scale (SAPS) and the Negative Symptoms Rating Scale (SANS) were used as data collection tools. Participants signed an informed consent form before the interview.

**Results:**

Data extraction is still ongoing in detailed style by principal authors. A description of the studies and the key findings will be presented.

**Conclusions:**

Reducing the risk of suicide in patients with schizophrenia is of vital importance. Awareness of the risks related to suicide may help reduce mortality rates in schizophrenia patients as in all patients. It is thought that the study’s results will be an important resource in knowing the risks related to suicide and determining the risk factors so that prevention studies can be initiated.

**Disclosure of Interest:**

None Declared

